# Environmental Applications of Chitosan Derivatives and Chitosan Composites

**DOI:** 10.3390/polym17192583

**Published:** 2025-09-24

**Authors:** Marián Lehocký

**Affiliations:** Centre of Polymer Systems, Tomas Bata University in Zlín, Trida Tomase Bati 5678, 760 01 Zlín, Czech Republic; lehocky@utb.cz

**Keywords:** chitosan, chitosan derivatives, chitosan composites, biopolymer composites, environmental remediation, pollutant removal, sustainable materials, circular economy

## Abstract

Chitosan, a naturally abundant and biodegradable biopolymer derived from chitin found in crustacean shells, has emerged as a promising material for addressing environmental challenges. Its reactive amino and hydroxyl groups enable diverse interaction mechanisms. This makes it effective for removing heavy metals, dyes, pharmaceuticals, and other contaminants from water. However, the limitations of native chitosan, such as poor solubility and mechanical strength, necessitate strategic modifications. This review comprehensively examines recent advances in chitosan derivatives and composites. It focuses on modern modification strategies, such as chemical, physical, and composite formation, that enhance stability, selectivity, and efficiency. It explores the design principles of high-performance composites. It also details the multifaceted mechanisms of pollutant removal, including adsorption, catalysis, membrane filtration, and flocculation. Critical practical challenges are critically assessed. These include scalability, regeneration, lifecycle sustainability, and real-world implementation. Furthermore, emerging trends are highlighted. These integrate circular economy principles, seafood waste valorization, and digital optimization through the use of artificial intelligence. By consolidating current knowledge, this review aims to bridge the gap between laboratory innovations and large-scale environmental applications. It guides the development of intelligent, scalable, and ecologically responsible solutions based on this remarkable biopolymer.

## 1. Introduction

**Figure 1:**
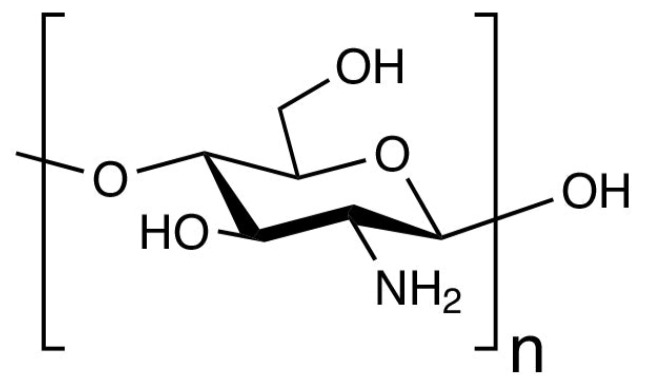
Chitosan structure.

The escalating global crisis of environmental pollution, fueled by the relentless discharge of industrial effluents, agricultural runoff, pharmaceutical residues, and the pervasive issue of improper waste management, has created an urgent and intensifying demand for remediation technologies. These technologies must be not only effective but also sustainable, efficient, and environmentally benign. Traditional synthetic polymers and chemical treatments, while historically prevalent, often fall short due to their non-renewable origins, potential for secondary pollution, and limited long-term ecological compatibility [1]. In this critical context, biopolymer-based materials, derived from renewable and often waste resources, have emerged as frontrunners in the quest for viable solutions [2]. Among these natural polymers, chitosan stands out with exceptional prominence. It is recognized for its remarkable versatility, abundance, and inherently eco-friendly characteristics [3,4]. As a deacetylated derivative of chitin [5], the second most abundant biopolymer on Earth after cellulose, chitosan is not merely a byproduct of nature’s vast structural inventory. It is a functional material with immense potential for addressing contemporary environmental challenges [6]. Its linear, cationic polysaccharide structure (Figure 1), composed of randomly distributed β-(1→4)-linked D-glucosamine and N-acetyl-D-glucosamine units, grants it a unique chemical identity. Of particular importance is the presence of reactive primary amino (–NH_2_) and hydroxyl (–OH) groups [7]. These functional moieties are the cornerstone of chitosan’s exceptional reactivity. They enable a diverse array of interaction mechanisms, such as electrostatic attraction, complexation, hydrogen bonding, ion exchange, and physical adsorption, that render it highly effective. It can capture and immobilize a broad spectrum of pollutants from aqueous environments, including heavy metal ions, organic dyes, pharmaceuticals, endocrine disruptors, and microbial contaminants [8,9,10].

The significance of chitosan is further amplified by its primary source: the vast quantities of crustacean shell waste generated by the global seafood industry. Annually, an estimated 6 to 8 million tons of shrimp, crab, and lobster shells are discarded. This presents a significant disposal challenge and environmental burden [11]. However, this waste stream simultaneously represents an underutilized reservoir of high-value raw material. The transformation of this waste into chitosan exemplifies a pivotal shift toward circular economy models. In these models, environmental liabilities are reimagined as resources for designing advanced functional materials [5,12]. While crustacean shells remain the dominant source, the exploration of alternative origins offers promising avenues for more sustainable and seasonally independent production. These include fungi such as *Aspergillus niger* and *Mucor rouxii*, insects like black soldier flies and crickets, and even bacterial cell walls [13,14]. The industrial journey from waste shell to functional biopolymer involves a well-defined, albeit traditionally energy-intensive, three-step process. First is demineralization using dilute acid to remove calcium carbonate. Second is deproteinization via alkaline treatment to eliminate proteins. Third is deacetylation under strong alkaline conditions (40–50% NaOH) at elevated temperatures to cleave acetyl groups from chitin [15]. The degree of deacetylation (DD), typically ranging from 55% to over 90%, is a crucial parameter defining the final properties. Higher DD values correlate with increased amino group content, enhanced cationic character, and improved reactivity. This is particularly true in acidic environments, where protonation renders the polymer soluble [16]. Molecular weight (MW), which varies widely from tens of thousands to several million Daltons, significantly influences viscosity, mechanical strength, solubility, and diffusion kinetics. These factors impact its applicability in various formats and conditions [17,18].

Despite its inherent advantages such as biocompatibility, non-toxicity, biodegradability, antimicrobial activity, film-forming ability, and mucoadhesive properties [19], native chitosan faces intrinsic limitations that constrain its direct, large-scale deployment in environmental systems. It is insoluble in neutral and alkaline pH conditions [20]. It is susceptible to acid hydrolysis [21]. It has low mechanical strength, a tendency to swell and disintegrate in aqueous media, limited surface area, and non-selective binding behaviour. These limitations necessitate strategic modifications to unlock its full potential. Consequently, extensive research has been devoted to overcoming these hurdles through a range of engineering strategies, including chemical derivatization, physical structuring, and composite formation. Chemical modifications have yielded derivatives like carboxymethyl chitosan (CMCS), which improves solubility across a wider pH range and exhibits ampholytic properties [22]. They have also produced quaternized chitosan, which maintains its positive charge even at neutral pH, enhancing its utility in diverse applications [23]. Another example is thiolated chitosan, which introduces redox activity and superior metal chelation capabilities, particularly for soft metals [24]. Physical processing techniques, such as cryostructuring, freeze-drying, and solution casting, enable the precise control of morphology and porosity. This allows the material to be tailored for specific functions [25]. However, perhaps the most impactful approach has been the development of composite materials, where chitosan is synergistically combined with various other components. Integration with inorganic fillers, such as diatomite, sepiolite, and layered silicates, enhances both the ion-exchange capacity and thermal stability of the material [26]. The incorporation of carbonaceous materials, such as biochar, graphene oxide, and carbon nanotubes, dramatically increases the surface area. It also introduces conductive pathways, thereby boosting both adsorption and catalytic capabilities [27]. Magnetic nanoparticles, such as Fe_3_O_4_, not only reinforce the structure but also facilitate easy separation after use. This is a crucial advantage for practical applications [28]. Combining chitosan with other biopolymers, such as alginate or lignin, further enhances mechanical properties and reduces costs [29,30]. These hybrid systems leverage the combined strengths of their constituents. They often yield performance that surpasses the sum of their parts. As demonstrated by composites capable of not only adsorbing but also catalytically reducing toxic species, such as Cr(VI), to less harmful forms, this approach yields enhanced performance [31].

The mechanisms by which chitosan-based materials remove pollutants are multifaceted and often work in concert. Adsorption, driven primarily by electrostatic interactions and complexation involving the amino and hydroxyl groups, is the dominant pathway [32]. It is particularly effective for capturing both anionic and cationic species under varying pH conditions [33]. Catalytic functionalities can be integrated into composites. This enables active degradation of pollutants beyond simple capture [34]. Chitosan’s film-forming ability and charge properties also make it an excellent candidate for membrane filtration. It offers antifouling characteristics beneficial for ultrafiltration and nanofiltration processes [35,36]. Furthermore, its natural cationic nature allows it to function as an effective flocculant. It aggregates negatively charged colloids in turbid water, providing a green alternative to synthetic coagulants [37]. While laboratory investigations have consistently demonstrated the efficacy of chitosan and its derivatives, the transition from bench-scale success to widespread real-world application is fraught with challenges. Issues related to scalability must be addressed. So too must maintaining performance under variable and often harsh environmental conditions. Efficient regeneration without causing secondary pollution is also critical. And ensuring overall lifecycle sustainability remains essential [31,38]. Traditional fabrication methods, such as lyophilization, are often energy-intensive. They are also challenging to scale up economically [39]. The economic viability of chitosan technology is closely tied to the cost-effective sourcing of raw materials. This makes the use of unrefined seafood waste more attractive than purified commercial grades [40]. Recognizing these challenges, the field is increasingly embracing green synthesis methods. Examples include enzymatic processing and microbial fermentation. These operate under milder conditions, reduce chemical consumption, and minimize hazardous waste generation. They align production more closely with sustainability goals [41]. Looking forward, the trajectory of chitosan technology is being further shaped by the integration of digital innovation. Artificial intelligence and machine learning are being harnessed to predict performance, optimize formulations, and enable real-time process monitoring. Concepts like digital twins and blockchain traceability are beginning to ensure transparency and efficiency across the entire value chain. This spans from waste sourcing to final disposal [42]. This confluence of material science, green chemistry, composite engineering, and digital technology underscores the evolving narrative of chitosan. It positions chitosan not just as a promising biopolymer, but as a cornerstone material for the development of scalable, intelligent, and ecologically responsible solutions. These are essential for global environmental protection in the 21st century [20].

Given the growing urgency of environmental degradation and the need for sustainable alternatives to synthetic polymers, this review provides a comprehensive synthesis of recent advances in chitosan-based materials [43]. By consolidating knowledge on its origin, structural characteristics, preparation methods, green synthesis routes, key derivatives, composite formulations, and functional properties, we aim to highlight the critical role of chitosan in addressing pressing ecological challenges [44]. The necessity of this review lies in bridging the gap between laboratory-scale innovations and real-world implementation. It is intended to guide future research toward scalable, intelligent, and ecologically responsible solutions. These solutions are grounded in one of nature’s most abundant and adaptable biopolymers: chitosan.

Based on the previous literature, review papers related to chitosan and its derivatives and composites primarily focus on their applications in the biomedical field, with some also exploring agricultural applications [45]. There is no comprehensive literature available that provides a systematic summary of recent knowledge related to the synthesis and environmental applications of chitosan and its derivatives, as well as composites. Therefore, this review aims to provide a comprehensive synthesis of recent advances in chitosan-based materials for environmental applications. We critically examine modern modification strategies, design principles of high-performance composites, the underlying mechanisms of pollutant removal, practical implementation barriers, and cutting-edge trends that integrate sustainability and digitalization. By consolidating current knowledge and identifying critical research gaps, this work aims to guide the development of scalable, intelligent, and ecologically responsible solutions for global environmental protection, based on one of nature’s most abundant and adaptable biopolymers: chitosan. Moreover, several applications of chitosan derivatives and chitosan-based composites are mentioned.

A comprehensive literature search was conducted in the Web of Science, Scopus, and PubMed databases using the following keywords and their combinations: “*chitosan*”, “*derivatives*”, and “*composites*.” The search covered publications from 2020 onward. Articles were included if they met the following criteria: (a) published in peer-reviewed journals, (b) written in English, and (c) focused on environmental applications. Exclusion criteria were: (i) conference abstracts or editorials, (ii) studies without sufficient experimental data, and (iii) publications unrelated to environmental applications.

## 2. Modification Strategies and Design Principles for Enhanced Environmental Performance of Chitosan

### 2.1. Modern Strategies for Modifying Chitosan to Enhance Stability, Selectivity, and Efficiency

The inherent functional richness of chitosan, stemming from its abundant primary amino (–NH_2_) and hydroxyl (–OH) groups, renders it a highly reactive biopolymer with significant potential for environmental applications [46]. However, native chitosan exhibits several limitations that restrict its practical utility, including poor solubility at neutral and alkaline pH levels [47], low mechanical strength [35], acid lability [29], swelling-induced disintegration in aqueous environments, a limited specific surface area [22,48], and non-selective interaction with pollutants [49]. To overcome these drawbacks and tailor chitosan for targeted remediation tasks, extensive research has been devoted to developing modern modification strategies. These approaches aim to enhance three critical performance parameters: stability, selectivity, and efficiency, thereby transforming chitosan from a naturally occurring polysaccharide into a high-performance, application-specific material [50].

One of the most widely employed methods to improve structural integrity and chemical resistance is chemical crosslinking [51]. This technique involves the formation of covalent bonds between chitosan chains using bifunctional agents such as glutaraldehyde, epichlorohydrin (ECH), genipin, or bisphenol A diglycidyl ether (BADGE) [51,52]. Crosslinked chitosan matrices exhibit enhanced durability under acidic conditions where unmodified chitosan would otherwise dissolve, making them suitable for prolonged use in industrial wastewater treatment systems [53]. For instance, ECH-cross-linked chitosan grafted with 2,4-dichlorobenzaldehyde has demonstrated high efficiency in Pb(II) ion removal due to increased availability of active binding sites and improved mechanical resilience [54]. The use of natural cross-linkers, such as genipin, is increasingly favored over synthetic alternatives due to their lower toxicity and greater biocompatibility, aligning with the principles of green chemistry and expanding their applicability in eco-sensitive domains [55].

In parallel, graft copolymerization represents a powerful strategy for enhancing both functionality and adsorption capacity [56]. By introducing vinyl monomers, such as acrylic acid, acrylamide, or polyethyleneimine (PEI), onto the chitosan backbone, researchers can precisely engineer the surface chemistry to target specific classes of contaminants [56]. PEI-functionalized chitosan aerogels, for example, display exceptional affinity for Cr(VI) anions even in the presence of competing ions, owing to the high density of protonated amine groups that facilitate strong electrostatic attraction [57]. Similarly, thio-urea-modified chitosan microspheres synthesized via microfluidic techniques exhibit rapid kinetics and high specificity for soft metal ions, such as Cd^2+^ and Hg^2+^, due to the formation of stable coordination complexes by sulphur-containing ligands [58]. These chemically engineered derivatives enable the selective capture of pollutants in complex effluents, significantly improving removal efficiency and regeneration potential.

Surface functionalization further refines chitosan’s performance by introducing molecular recognition sites that enhance selectivity toward target pollutants [59]. Reactions such as Schiff base formation, where aldehydes like salicylaldehyde react with chitosan’s amino groups to form imine linkages (-C=N-), can create selective chelating centres for transition metals [60]. Functionalization with amino acids, such as 4-aminobenzoic acid, adds carboxylate groups that increase coordination capacity with cationic species like Cu^2+^ and Ni^2+^ [61]. Additionally, integration of ionic liquids or surfactant moieties imparts dual functionality, combining hydrophobic interactions with electrostatic forces to improve dye adsorption, as observed in systems designed for tartrazine removal [62]. Such targeted modifications transform chitosan from a broadly reactive biopolymer into a precision material capable of discriminating between structurally similar contaminants.

An efficient approach involves the creation of composite systems, where chitosan is combined with inorganic, carbonaceous, or polymeric materials to achieve synergistic enhancements [63]. The synthesis of organic–inorganic composites has proven successful in producing materials with superior properties compared to individual components. For example, incorporating silica-based materials such as diatomite, sepiolite, or nanosilica not only increases porosity and ion-exchange capacity but also stabilizes the chitosan matrix against swelling and degradation [64]. Studies have shown that chitosan–nanosilica composites (ChNS) and chitosan–silica gel composites (ChSG), prepared through mechanosorption and geometric modification of silica, exhibit significantly enhanced adsorption efficiency, especially under acidic conditions (pH = 2), highlighting the importance of stabilizing chitosan in aggressive environments [65].

Similarly, integration with carbon-based nanomaterials, including graphene oxide (GO), carbon nanotubes (CNTs), and biochar, not only improves mechanical strength and electrical conductivity but also dramatically increases specific surface area and electron transfer rates, which are beneficial for both adsorption and photocatalytic degradation processes [66]. Magnetic nanoparticles, such as Fe_3_O_4_ or CoFe_2_O_4_, are embedded within chitosan matrices to combine excellent adsorption properties with the ease of magnetic separation, enabling efficient recovery and reuse without the need for filtration or centrifugation [30,67]. Furthermore, hybrid systems like MOF/chitosan composites leverage the enormous surface area and tunable pores of metal–organic frameworks with chitosan’s film-forming ability and processability, yielding highly efficient adsorbents for heavy metals such as Pb(II) and U(VI) [68].

Physical modifications also play a crucial role in overcoming the intrinsic limitations of chitosan. Techniques such as solution casting, granulation, freeze-drying, and cryostructuring enable the transformation of chitosan into various morphologies, including beads, films, flakes, microspheres, aerogels, and membranes, each tailored for specific applications [69]. These physical forms help mitigate defects such as low porosity and low specific surface area, allowing better exposure of functional groups during the adsorption process [70]. For instance, cryostructured composites made from chitosan and silicate minerals exhibit highly interconnected porous networks that are ideal for pollutant uptake. During freezing, ice crystals formed act as templates for macropores, facilitating the rapid diffusion of contaminants into the bulk material [71].

Moreover, chemical alterations such as quaternization have been shown to broaden the solubility spectrum of chitosan from exclusively acidic media (pH < 6) to neutral and basic conditions (pH 4–9) [32]. Quaternary ammonium groups, either introduced directly onto the chitosan backbone or via spacers, impart a permanent positive charge regardless of solution pH, enabling operation across a broader range of environmental conditions [72]. A reported increase in the aqueous solubility of chitosan, up to 80 mg/mL, was achieved through quaternization, demonstrating its potential for formulation in diverse aqueous systems [73].

Another emerging trend is the amplification and modification of complex systems through the incorporation of multifunctional additives. Adding components such as montmorillonite, polycaprolactone, or essential oils not only enhances mechanical and barrier properties but also introduces new functionalities like antimicrobial activity and antioxidant release [74]. Bio-polymer/clay composites, for example, have drawn considerable attention due to the synergistic enhancement of physical and chemical properties compared to pure chitosan [75]. The inclusion of varying amounts of chitosan enables the fine-tuning of composite characteristics, which is crucial for future biomedical and environmental applications [76].

Finally, recent studies emphasize the need to balance the benefits of modification with cost-effectiveness and reusability. While crosslinking improves stability, it may reduce the number of accessible functional groups, thereby affecting adsorption capacity [77]. Therefore, finding an optimal equilibrium between structural reinforcement and functional availability is critical for maximizing overall performance. Future development should focus on exploring more economical and convenient preparation methods, reducing material costs, improving reuse rates, and minimizing secondary pollution from regeneration processes [78].

In summary, modern strategies for modifying chitosan encompass a broad spectrum of physical and chemical techniques, including crosslinking, grafting, compositing, and quaternization, all aimed at enhancing stability, selectivity, and efficiency. By rationally designing these modifications based on pollutant characteristics and application requirements, researchers are creating next-generation chitosan-based materials that are not only scientifically advanced but also practically viable for real-world environmental remediation. In terms of enhanced stability, the literature cited above explains, that stability of crosslinked chitosan microspheres in acidic conditions was confirmed through repeated adsorption–desorption cycles, with studies reporting over 85% retention of adsorption capacity after five cycles, indicating strong structural integrity under operational conditions. Thermal stability improvements were documented via TGA analysis, where chitosan-clay or chitosan-nanoparticle composites showed decomposition onset temperatures 30–50 °C higher than native chitosan, reflecting enhanced thermal resistance. Mechanical stability was evaluated in composite films, with one study reporting a 2.5-fold increase in tensile strength for chitosan-cellulose nanofiber blends compared to pure chitosan films. Swelling behavior and pH-responsive stability were assessed in situ, showing minimal volume change over multiple cycles, confirming three-dimensional stability of the polysaccharide-based composites.

### 2.2. Fundamental Design Principles Guiding the Development of High-Performance Chitosan Composites

The development of high-performance chitosan composites is rooted in a systematic and multidisciplinary approach that integrates materials science, polymer chemistry, and environmental engineering [79]. These composites are not merely physical mixtures but rationally engineered systems designed to overcome the inherent limitations of native chitosan, such as poor mechanical strength, low thermal stability, acid solubility, swelling-induced disintegration, and limited surface area [80], while amplifying its advantageous properties, including biocompatibility, biodegradability, cationic character, and rich functional group chemistry [81]. The successful design of these advanced materials relies on several fundamental principles: interfacial compatibility and adhesion, morphological control and architectural engineering, functional synergy between components, stabilization through crosslinking, and application-driven tailoring [82].

A cornerstone of composite design is achieving strong interfacial compatibility and adhesion between the chitosan matrix and the reinforcing or functional filler. Without adequate bonding, phase separation, agglomeration, and poor stress transfer can occur, leading to structural weaknesses and reduced performance [83]. To enhance compatibility, fillers such as diatomite, sepiolite, nanosilica (NS), silica gel (SG), graphene oxide (GO), metal oxides (e.g., ZnO, Fe_3_O_4_, TiO_2_), or clay minerals (e.g., montmorillonite, MMT; natural zeolite, NZ) are often surface-modified or integrated via chemical linkers [84]. For instance, studies have demonstrated that chitosan–nanosilica composites (ChNS) and chitosan–silica gel composites (ChSG) synthesized through mechanosorption and geometric modification of silica exhibit significantly improved adsorption efficiency, particularly under acidic conditions (pH = 2), where unmodified chitosan would typically dissolve [49,85]. The use of cross-linkers, such as glutaraldehyde (GA), further strengthens the interface by forming covalent bonds between chitosan chains and the filler surface, as evidenced in composites like ChNS_1_GA and ChSG_1_GA, which exhibit enhanced structural integrity and resistance to degradation [86].

Closely tied to interfacial engineering is the principle of morphological control and architectural design [87]. The physical form and internal architecture of a composite directly influence its functional performance, especially in applications involving mass transfer, such as adsorption or catalysis [88]. Techniques such as solution casting, drop granulation, freeze-drying, cryostructuring, electrospinning, and hot-water stretching enable precise manipulation of porosity, pore size distribution, specific surface area, and mechanical resilience [89]. For example, cryostructured composites prepared from chitosan and silicate minerals exhibit highly interconnected porous networks that facilitate the rapid diffusion of pollutants into the bulk material, making them ideal for the efficient uptake of persistent pharmaceuticals, such as carbamazepine (CBZ) and ciprofloxacin (CIP) [59,90]. Similarly, scanning electron microscopy (SEM) analyses of chitosan–SiO_2_ composites reveal well-dispersed morphologies with smooth surfaces and minimal agglomeration, indicating good integration and homogeneity at the microscale [91]. In fibre-based systems, wet-spinning followed by immersion in dopamine lye has been shown to create a protective polydopamine (PDA) coating around chitosan fibres, significantly improving their mechanical properties and enabling potential use in biomedical and load-bearing applications [92].

Another critical design principle is the establishment of functional synergy between components, where each constituent contributes uniquely to the overall capability of the system [93]. Rather than simply combining materials, modern composites are engineered so that the whole performs better than the sum of its parts [94]. Magnetic nanoparticles such as Fe_3_O_4_ or CoFe_2_O_4_ are embedded within chitosan matrices not only to reinforce mechanical properties but also to enable easy magnetic separation after pollutant binding, a feature essential for practical wastewater treatment operations [95]. Photocatalytic materials, such as TiO_2_ or ZnO, introduce light-responsive degradation capabilities, allowing chitosan composites to act not only as passive adsorbents but also as active agents in breaking down organic dyes, including methyl orange and reactive red 120, under UV irradiation [96]. Antimicrobial functionalities are similarly enhanced by incorporating silver nanoparticles, curcumin, or rosemary extract, which work synergistically with chitosan’s intrinsic biocidal properties [87,97]. Hybrid systems, such as chitosan-alginate@Fe/Mn mixed oxide nanocomposites, exemplify this synergy by first reducing toxic Cr(VI) to less harmful Cr(III) through redox reactions and then immobilizing the resulting cation through complexation with the amino groups of chitosan [51].

Stabilization through crosslinking plays a decisive role in determining the durability and operational lifespan of chitosan composites [98]. Crosslinking agents such as glutaraldehyde, epichlorohydrin (ECH), genipin, vanillin, or tripolyphosphate (TPP) create a three-dimensional network that resists dissolution in aqueous environments, particularly under acidic conditions [52]. This stabilization is crucial for maintaining structural integrity during prolonged exposure to industrial effluents [99]. Moreover, natural cross-linkers, such as genipin, offer lower cytotoxicity compared to synthetic alternatives, making them suitable for eco-friendly and biomedical applications [100]. Studies on porous chitosan composites stabilized using various methods have shown that crosslinking not only enhances mechanical strength but also affects pycnometric density and specific surface area, which are vital parameters for both implant materials and adsorbents [101].

Ultimately, the design of chitosan composites should be guided by the intended application, ensuring that the material meets the functional, economic, and sustainability criteria [95]. For environmental remediation, composites are optimized for high porosity, selectivity toward target pollutants (e.g., Pb(II), Cd(II), As(V), and anionic dyes), and regenerability [2,102]. In biomedical contexts, emphasis is placed on cytocompatibility, controlled release, antibacterial action, and mechanical mimicry of natural tissues. For food packaging, barrier properties against moisture and gases, as well as antioxidant and antimicrobial functions, are prioritized [103]. The inclusion of varying amounts of chitosan enables the fine-tuning of composite characteristics, for example, adjusting the chitosan/carbon ratio in aerogel composites (Car/CSAs) to maximize methylene blue (MB) removal efficiency [104]. Similarly, in dental restorative materials, the addition of nano-chitosan to adhesive and flowable composite resins has been evaluated for its impact on mechanical phenomena, solubility, and antimicrobial activity, demonstrating how even minor modifications can yield significant functional improvements [105].

Thus, the rational design of high-performance chitosan composites follows a holistic framework that balances interfacial adhesion, structural architecture, functional integration, chemical stabilization, and end-use requirements. By leveraging these fundamental principles, researchers are creating innovative, sustainable, and multifunctional materials that fully exploit the potential of one of nature’s most versatile biopolymers, such as chitosan, paving the way for scalable and environmentally responsible solutions across diverse fields.

### 2.3. Key Mechanisms Underlying Pollutant Removal via Adsorption, Catalysis, Membrane Filtration, and Flocculation

The effectiveness of chitosan-based materials in environmental remediation stems from their ability to engage multiple mechanisms for pollutant removal, each operating through distinct physicochemical pathways. These mechanisms, such as adsorption, catalysis, membrane filtration, and flocculation, which are not mutually exclusive but often function synergistically within engineered composites, enable efficient, selective, and scalable treatment of complex wastewater streams [106]. The integration of these processes allows for the simultaneous targeting of diverse contaminants, including heavy metals, organic dyes, pharmaceuticals, radionuclides, and microbial agents [107].

Adsorption is widely recognized as one of the most effective, economical, and environmentally friendly methods for removing pollutants from aqueous systems [108]. It involves the accumulation of contaminant molecules (adsorbates) onto the surface of a solid material (adsorbent) through various intermolecular forces [109]. Chitosan and its derivatives are particularly effective adsorbents due to their abundant functional groups, primarily primary amino (–NH_2_) and hydroxyl (–OH) groups, which participate in multiple interaction mechanisms [9,29,110]. Under acidic conditions (pH < 6), the protonation of amino groups generates positively charged –NH_3_^+^ sites that facilitate strong electrostatic attraction with anionic species such as chromate [Cr(VI)], arsenate [As(V)], sulfate dyes, uranyl ions [UO_2_^2+^], and other oxyanions [51,61,63]. For cationic pollutants like Pb(II), Cd(II), Cu(II), Ni(II), and Zn(II), adsorption occurs via complexation or chelation, where metal ions coordinate with lone electron pairs on nitrogen and oxygen atoms, forming stable five- or six-membered rings [111,112]. This mechanism is further enhanced in modified chitosan systems, such as those incorporating carboxylate, phosphate, or thiol groups, which increase the number of available binding sites and improve selectivity [113].

In addition to electrostatic and coordination interactions, hydrogen bonding, van der Waals forces, and π–π stacking contribute to the overall adsorption process, particularly when chitosan is combined with carbonaceous materials such as graphene oxide (GO) or biochar [114]. The presence of oxygen-containing functional groups on GO enhances its affinity for both organic and inorganic pollutants, making it highly effective for the adsorption of radionuclides such as Eu(III), Pu(IV), and Am(III) [115,116]. Furthermore, ion exchange plays a significant role in composites containing inorganic fillers such as zeolites, layered silicates, or hydroxyapatite, where target metal ions replace mobile counterions (e.g., Ca^2+^, Na^+^) during the uptake process [117].

The intraparticle diffusion model reveals that the adsorption process typically proceeds through three distinct stages: bulk diffusion, film diffusion, and pore diffusion [118]. Bulk diffusion refers to the transport of pollutants from the bulk solution to the external surface of the adsorbent. Film diffusion involves the movement of contaminants across the boundary layer surrounding the particle, while pore diffusion governs the penetration of adsorbates into the internal porous network. In diatomite-chitosan composites, for example, physical adsorption, electrostatic interaction, and surface complexation collectively enhance vanadium [V(V)] removal efficiency [119]. Notably, some chitosan-based materials also exhibit reductive adsorption, where functional groups such as hydroxyl oxygen atoms reduce toxic V(V) to less harmful V(IV), thereby combining chemical transformation with immobilization [120].

Catalysis represents an advanced mode of pollutant removal that surpasses passive capture, enabling the active degradation of persistent organic pollutants [121]. While native chitosan is not inherently catalytic, it serves as an excellent support matrix for catalytically active species such as metal nanoparticles (e.g., Fe/Mn mixed oxides, TiO_2_, ZnO), enzymes, or conjugated polymers [122]. Chitosan-alginate@Fe/Mn mixed oxide nanocomposites, for instance, demonstrate high efficiency in reducing toxic Cr(VI) to less hazardous Cr(III) through redox reactions, followed by immobilization via complexation with chitosan’s amino groups [40,123]. This dual functionality, simultaneous reduction and adsorption, significantly improves detoxification outcomes. Similarly, chitosan-supported photocatalysts, such as TiO_2_ or SnO_2_, can degrade organic dyes under UV or visible light irradiation, leveraging the biopolymer’s film-forming ability and dispersion capacity to prevent nanoparticle aggregation [124]. In more sophisticated systems, chitosan-based aerogels or cryogels act as hosts for noble metal catalysts, facilitating reactions such as the oxidation of phenolic compounds or the breakdown of antibiotics like tetracycline without generating harmful byproducts [125].

Membrane filtration utilizing chitosan-based membranes has gained increasing attention due to the polymer’s excellent film-forming properties, tunable porosity, and antimicrobial characteristics [126]. These membranes operate through a combination of size exclusion, charge repulsion, and adsorptive retention, depending on the membrane architecture and surface chemistry [127]. Composite membranes made from chitosan and graphene oxide (CSGO), for example, exhibit improved flux, reduced fouling, and high rejection rates for dyes and heavy metals, making them suitable for greywater treatment and non-potable reuse applications [128]. The incorporation of GO increases mechanical strength and specific surface area, while chitosan contributes positive surface charge and biofouling resistance. Moreover, pH-responsive swelling behavior enables smart membrane operation, where pore size and permeability can be modulated by adjusting the solution pH, making them ideal for selective separation processes [129]. In some configurations, such as mixed matrix membranes (MMMs) or membrane adsorbers, embedded adsorbent particles (e.g., activated carbon, MOFs) provide additional binding sites, transforming the membrane into a multifunctional platform capable of both filtration and adsorption [130,131].

Flocculation is another vital mechanism in which chitosan acts as a natural cationic flocculant, neutralizing negatively charged colloidal particles and suspended solids in wastewater [132]. Upon addition to contaminated water, chitosan chains adsorb onto particle surfaces, compressing the electrical double layer and promoting aggregation through charge neutralization and polymer bridging [133]. This results in the formation of larger flocs that settle rapidly under gravity, facilitating easy removal of turbidity, organic matter, and even pathogens [134]. The effectiveness of chitosan as a flocculant depends on its degree of deacetylation, molecular weight, and solution pH [23,51,135]. High-DD chitosan offers more amino groups for charge interactions, while a higher molecular weight enhances chain extension and inter-particle bridging capability [136]. Unlike synthetic flocculants such as polyacrylamide, chitosan is biodegradable and non-toxic, making it an environmentally favourable alternative [135]. It has been successfully used in treating textile effluents, food processing wastewaters, and algal blooms, often outperforming conventional coagulants in terms of reducing sludge volume and improving the clarity of treated water [137].

The synergy between these mechanisms is increasingly exploited in multifunctional chitosan composites. For example, a single system may combine adsorptive capture, catalytic degradation, and magnetic separation, enabling complete pollutant elimination with minimal operational complexity [138]. The design of such integrated platforms relies on a deep understanding of interfacial phenomena, mass transfer dynamics, and reaction kinetics, all of which are informed by advanced characterization techniques and modelling approaches [139].

In summary, chitosan-based materials employ a multifaceted approach to pollutant removal, integrating adsorption, catalysis, membrane filtration, and flocculation in a manner that is both complementary and synergistic [109]. By rationally engineering the chemical structure, morphology, and functional architecture of these composites, researchers can tailor their performance to specific environmental challenges. This mechanistic versatility underscores chitosan’s role as a cornerstone material in the development of next-generation, sustainable water purification technologies [140].

Thus, processes like adsorption, catalysis, membrane filtration, and flocculation are not isolated but operate synergistically within engineered composites to address complex, multi-pollutant wastewater streams. For instance, chitosan’s abundant amino and hydroxyl groups enable simultaneous adsorption of heavy metals and organic dyes through electrostatic interactions and complexation, while its film-forming capacity allows it to serve as a selective layer in mixed matrix membranes, enhancing separation efficiency and mitigating fouling through surface charge repulsion. The mechanism begins with electrostatic adsorption under acidic conditions (pH 2–5), where the amino groups (–NH_2_) of chitosan are protonated to –NH_3_^+^, enabling strong attraction to anionic Cr(VI) species such as HCrO_4_^−^ or Cr_2_O_7_^2−^:

Chitosan-NH_2_ + H^+^ → Chitosan-NH_3_^+^

Chitosan-NH_3_^+^ + HCrO_4_^−^ → Chitosan-NH_3_^+^⋯HCrO_4_^−^

Following adsorption, the reduction of highly toxic Cr(VI) to less harmful Cr(III) occurs, primarily mediated by the electron-donating nature of the –NH_2_/–NH_3_^+^ groups:

3Chitosan-NH_2_ + HCrO_4_^−^ + 7H^+^ → Cr^3+^ + 3Chitosan-NH_3_^+^ + 4H_2_O

Finally, the generated Cr_3_^+^ ions undergo complexation with unprotonated amino and hydroxyl groups of chitosan, forming stable coordination bonds that prevent leaching:

Cr^3+^ + 6Chitosan-OH/NH_2_ → [Cr(Chitosan)_6_]^3+^

This three-stage synergy—where physical concentration, chemical detoxification, and immobilization occur in tandem—is a key advantage of chitosan over conventional adsorbents.

When combined with catalytic nanoparticles (e.g., TiO_2_, Fe_3_O_4_, ZnO), chitosan composites can not only capture but also degrade pollutants via photocatalytic or Fenton-like reactions, transforming persistent organics into less harmful byproducts. This integration of capture and destruction reduces the risk of secondary pollution and minimizes the need for adsorbent regeneration. Furthermore, the cationic nature of chitosan promotes its role as a green flocculant, bridging colloidal particles and facilitating their aggregation and removal.

### 2.4. Practical Challenges Related to Scalability, Regeneration, Lifecycle Sustainability, and Real-World Implementation

Despite the extensive research and promising laboratory-scale performance of chitosan-based materials for environmental applications, their transition from benchtop innovation to widespread industrial and municipal deployment faces a series of interconnected practical challenges [20]. These include issues related to the scalability of synthesis, regeneration, and reusability, as well as lifecycle sustainability and real-world operational constraints, all of which must be addressed to ensure that chitosan composites are not only scientifically practical but also economically viable, environmentally sound, and technically feasible in complex, dynamic environments [141].

One of the most significant barriers to large-scale adoption is the scalability of fabrication methods. Many high-performance chitosan composites are synthesized using techniques such as freeze-drying, electrospinning, layer-by-layer assembly, or cryostructuring, which offer excellent control over morphology, porosity, and functional integration [22,47,142]. However, these processes are inherently complex to scale up due to high energy demands, batch-to-batch variability, and low throughput. For example, aerogels and cryogels with ultra-high surface areas and 3D porous networks demonstrate exceptional adsorption capacity in controlled settings; however, their production requires specialized equipment, such as lyophilizers or supercritical CO_2_ dryers, making them impractical for continuous manufacturing at industrial levels [143]. Similarly, graft copolymerization, magnetic nanoparticle incorporation, or surface functionalization often involves multi-step procedures, toxic crosslinking agents (e.g., glutaraldehyde), or stringent reaction conditions that complicate process standardization and raise concerns regarding safety and waste management [144]. To enable mass production without compromising performance, there is an urgent need for simple, reproducible, and environmentally friendly fabrication routes, such as solution casting, drop granulation, dip-coating, or air-spray deposition, that can be seamlessly integrated into existing water treatment infrastructure [145].

Closely linked to scalability is the issue of mechanical and chemical stability under real operating conditions. In practical wastewater treatment scenarios, chitosan-based materials must endure fluctuating pH levels, high ionic strength, microbial activity, abrasive flow dynamics, and prolonged exposure to organic solvents or surfactants [136,146]. Native chitosan dissolves readily in acidic environments (pH < 6), severely limiting its applicability in effluents rich in mineral acids or organic acids commonly found in textile, mining, or electroplating industries [51]. Although crosslinking improves acid resistance, it may reduce the availability of active binding sites and slow down adsorption kinetics [77,147]. Furthermore, repeated swelling and deswelling cycles during use and regeneration can lead to structural fatigue, cracking, or disintegration over time [29]. Field trials have shown that while chitosan-diatomite, chitosan-sepiolite, or chitosan-alginate composites perform well initially, their efficiency declines after extended exposure to complex matrices containing oils, suspended solids, or competing ions [148]. Therefore, developing mechanically robust, chemically stable, and fouling-resistant composites remains a critical challenge for long-term implementation [149]. For understanding these processes, the most important parameters are mentioned related to the comparative life cycle impact of chitosan-based materials based on their source and modification method in Table 1.

Another crucial factor affecting the economic viability of chitosan-based systems is regeneration and reusability. An ideal adsorbent should not only exhibit high removal capacity but also be efficiently regenerated and reused over multiple cycles to minimize material consumption and operational costs [150]. Several studies confirm that certain chitosan composites can retain significant functionality after regeneration. For instance, PEI-functionalized chitosan hydrogels maintained about 66% of their initial adsorption capacity after four cycles when eluted with 1 M HCl, indicating good recovery potential [151]. However, regeneration protocols often require large volumes of strong acids, bases, or organic solvents, raising concerns about secondary pollution, resource consumption, and handling hazards [152]. The necessity for extensive washing, where 1 g of sorbent may require up to 0.3 L of water, further questions the ecological justification of regeneration, especially in water-scarce regions [153]. As a result, alternative end-of-life strategies are being explored, such as converting spent sorbents into construction materials, soil amendments, or catalyst supports, thereby shifting from circular reuse to integrated valorisation [154].

From a broader perspective, the lifecycle sustainability of chitosan composites must be evaluated holistically. While chitosan itself is derived from renewable sources, primarily seafood waste, and is biodegradable, the addition of synthetic polymers, nanoparticles, or non-biodegradable fillers (e.g., graphene oxide, carbon nanotubes, polyvinyl alcohol) can alter its environmental footprint [155]. Energy-intensive extraction and deacetylation processes, involving concentrated NaOH and HCl, contribute to greenhouse gas emissions and generate alkaline/acidic wastewater, thereby undermining the overall green credentials of the final product [31]. A comprehensive lifecycle analysis (LCA) should therefore assess all stages from raw material sourcing and processing to application, regeneration, and final disposal or recycling, to ensure net environmental benefit [156]. Emphasis should be placed on minimizing chemical usage, reducing energy input, and maximizing recyclability and biodegradability across the entire value chain [157].

Economic feasibility remains a key factor in determining real-world adoption. Although it is a byproduct of the fishing industry, purified chitosan can be more expensive than conventional adsorbents, such as activated carbon or ion-exchange resins [158]. Cost-effectiveness improves significantly when unrefined seafood waste (e.g., shrimp shells, crab exoskeletons) is used directly as feedstock, or when composites are designed for dual functions, such as simultaneous oil-water separation and heavy metal capture, or combined flocculation and disinfection [159]. Integration with existing technologies, such as fixed-bed columns, membrane modules, or transformer oil regeneration units, also enhances feasibility [31]. Pilot-scale testing in actual industrial effluent streams has demonstrated promising results, showing that chitosan composites can extend equipment lifespan, reduce sludge volume, and lower hazardous waste generation [160]. Moreover, the use of composite adsorbents for regenerating insulating transformer oil contributes to both environmental protection and energy resilience by minimizing the need for frequent oil replacement and disposal [161].

Despite their promise, chitosan-based materials face practical challenges in real-world applications. Fouling from organic matter, suspended solids, or biofilms reduces efficiency and increases maintenance in continuous systems. Regeneration often requires strong acids or bases, leading to secondary pollution, polymer degradation, and high water and chemical consumption up to 0.3 L per gram of adsorbent, raising economic and ecological concerns. Moreover, regulatory frameworks such as the EU’s target for 100 percent reusable, recyclable, or compostable plastic packaging by 2025 demand that biopolymers like chitosan meet strict standards for compostability and non-toxicity. While chitosan aligns with circular economy goals due to its biodegradability and waste-derived origin, insufficient data on nanoparticle leaching and ecotoxicity hinder regulatory approval. Future development must therefore focus on fouling-resistant designs, green regeneration protocols, and standardized lifecycle assessments to ensure both performance and compliance.

In summary, while chitosan-based materials hold immense promise for sustainable environmental remediation, their widespread implementation requires overcoming multifaceted challenges beyond laboratory performance [162]. Addressing scalability, ensuring robustness under variable conditions, optimizing regeneration protocols, conducting rigorous lifecycle assessments, and aligning with circular economy principles are essential steps toward transforming these innovative materials from scientific curiosities into practical, economically viable, and environmentally responsible solutions for global pollution control [163]. Future research must focus on bridging the gap between scientific potential and real-world impact, ensuring that the full benefits of chitosan technology are realized not only in journals but also in rivers, soils, industries, and communities worldwide [164].

### 2.5. Emerging Trends: Circular Economy Integration, Seafood Waste Valorisation, and Digital Optimization Using Artificial Intelligence

The field of environmental remediation using chitosan-based materials is undergoing a profound transformation, driven by global imperatives for sustainability, resource efficiency, and technological innovation. Among the most significant emerging trends are the integration of chitosan technology within circular economy frameworks, the systematic valorisation of seafood processing waste as a primary feedstock, and the application of digital tools, particularly artificial intelligence (AI) and machine learning (ML) for material design, process optimization, and system monitoring. These interconnected advancements are shifting the paradigm from linear, extractive models of production to regenerative, intelligent systems that align scientific progress with ecological responsibility [165,166].

A cornerstone of this evolution is the integration of chitosan into circular economy principles, a model that emphasizes closed-loop systems, waste minimization, and the continuous reuse of resources [167]. Chitosan exemplifies this approach by transforming an abundant industrial byproduct, such as crustacean shells from fisheries and aquaculture, into high-value functional materials for water treatment, food packaging, biomedical applications, and agriculture [168]. Globally, over 6-8 million tons of shellfish waste are generated annually, often ending up in landfills or being incinerated, which contributes to environmental pollution and greenhouse gas emissions [169]. By redirecting this waste stream into biorefineries for chitin and chitosan extraction, industries can simultaneously reduce their ecological footprint and create new economic opportunities [170]. Furthermore, end-of-life strategies for spent chitosan composites are increasingly designed with circularity in mind: exhausted adsorbents loaded with heavy metals can be processed for metal recovery via acid elution and precipitation. At the same time, biodegradable matrices can be composted or repurposed as soil conditioners, thereby closing nutrient loops and supporting sustainable agriculture [154]. The European Union’s new circular economy action plan explicitly promotes biopolymers, such as chitosan, as revolutionary alternatives to conventional synthetic polymers that persist in ecosystems, underscoring the policy-level recognition of their transformative potential [171].

Closely linked to this shift is the valorisation of seafood waste, which has emerged as a critical driver of sustainable chitosan production. Traditionally, shrimp, crab, lobster, and other crustacean shells were considered low-value residues requiring costly disposal. However, recent innovations in green extraction technologies, such as enzymatic deproteinization, microbial fermentation, and mild demineralization using organic acids, have enabled the more efficient and eco-friendly recovery of high-purity chitin and chitosan [172]. Enzymatic methods, in particular, offer significant advantages over conventional acid-alkali treatments by reducing energy consumption, chemical usage, and wastewater generation [173]. For instance, proteolytic enzymes such as alcalase or papain selectively break down proteins without damaging the chitin structure, yielding higher-quality products with a lower environmental impact [174]. Moreover, studies involving *Bacillus* sp. XT7 and other microorganisms demonstrate how fermentative processes can integrate deproteinization and demineralization steps under mild conditions, further enhancing process sustainability [175]. This trend extends beyond crustaceans; research on black tiger shrimp (*Penaeus monodon*) heads has demonstrated the use of integrated approaches for recovering both protein hydrolysate and chitosan, moving toward zero-waste biorefinery models [176]. Similarly, fungal biomass and insect exoskeletons are being explored as alternative sources, diversifying supply chains and reducing pressure on marine ecosystems [177].

Parallel to these advancements in bioresources, a new frontier is emerging through the application of artificial intelligence (AI), machine learning (ML), and data-driven modeling in the development and deployment of chitosan-based systems [178]. Traditional approaches to designing chitosan composites rely heavily on empirical experimentation, a time-consuming and resource-intensive process that is prone to inefficiencies [22,179]. In contrast, AI-driven methodologies enable predictive analytics, real-time monitoring, and adaptive control across the entire lifecycle of chitosan materials [180]. Machine learning algorithms have been successfully applied to optimize biochar synthesis parameters, accurately forecasting yield and performance based on feedstock composition, pyrolysis temperature, and activation conditions, which is an approach directly transferable to chitosan composite fabrication [181]. Similarly, neural networks and regression models can simulate adsorption isotherms, kinetic behavior, and mechanical properties under varying pH, ionic strength, and contaminant concentrations, allowing researchers to identify optimal formulations without the need for exhaustive laboratory testing [182].

Advanced computational techniques, such as adaptive neuro-fuzzy inference systems (ANFIS) and artificial neural networks (ANNs), are proving particularly effective in modeling complex adsorption processes [183]. For example, ANNs have been used to predict the removal efficiency of methylene blue dye using modified clay, outperforming traditional response surface methodology (RSM) in accuracy and flexibility [184,185]. These models can account for nonlinear interactions between variables such as contact time, initial concentration, pH, and adsorbent dosage, which are difficult to capture with conventional statistical methods [186]. Furthermore, AI integration extends to operational decision-making in wastewater treatment plants where chitosan-based adsorbents are deployed. Intelligent sensors, combined with data analytics platforms, can monitor effluent quality in real-time, dynamically adjusting dosage rates, contact times, and regeneration cycles to maximize efficiency and minimize chemical consumption [160].

Digital twins, as virtual replicas of physical treatment systems, can simulate long-term performance, forecast maintenance needs, and evaluate the environmental impact of different operational scenarios [187]. When coupled with blockchain-enabled traceability systems, these technologies ensure transparency across the chitosan supply chain, from raw material sourcing to final disposal [188]. This level of digital integration not only accelerates innovation but also enhances accountability and public trust in green technologies [189].

Moreover, AI is playing a crucial role in optimizing emerging hybrid systems such as magnetic nanocomposites, photocatalytic membranes, and stimuli-responsive hydrogels [190]. For instance, multi-criteria decision-making tools, such as fuzzy AHP-TOPSIS, are being used to select the best colour removal process using carbon-based adsorbents under real-world conditions, balancing technical performance with economic feasibility [191]. Similarly, AI-assisted analysis of georeferenced medical and viral dispersion data is paving the way for integrated epidemic control systems, demonstrating how digital infrastructure can support broader sustainability goals beyond environmental remediation [192].

In summary, the convergence of circular economy thinking, seafood waste valorisation, and AI-driven optimization represents a paradigm shift in the field of chitosan science [141]. These trends collectively move the discipline away from isolated, linear research efforts toward integrated, intelligent, and sustainable solutions [44]. As global pressures on natural resources intensify, the ability to transform waste into high-performance materials using data-informed, eco-conscious methods will be essential, not only for advancing scientific knowledge but also for building resilient and regenerative environmental systems [193]. The continued evolution of chitosan technology in this direction promises to position it at the forefront of the green materials revolution, offering scalable, innovative, and sustainable solutions to some of the world’s most pressing environmental challenges [20].

## 3. Practical Environmental Applications of Chitosan Derivatives and Composites

### 3.1. Water Treatment

Chitosan and its derivatives have emerged as pivotal materials in modern water treatment technologies due to their exceptional physicochemical properties, biocompatibility, and sustainable origin. Derived from the deacetylation of chitin, abundant in crustacean shells, chitosan is a linear cationic polysaccharide with reactive amino and hydroxyl groups that enable multifunctional interactions with a wide range of aquatic pollutants [194]. Its application spans heavy metal removal, dye decolorization, organic contaminant adsorption, and microbial control, making it one of the most versatile biopolymers for environmental remediation [195].

#### 3.1.1. Heavy Metal Removal

The removal of toxic heavy metals such as Pb(II), Hg (II), Cd(II), Cu(II), Cr(VI), As(V), and Zn(II) from aqueous systems is a critical aspect of water purification, particularly in industrial effluents and contaminated groundwater [196]. Chitosan-based materials excel in this domain through several mechanisms, including coordination, ion exchange, and electrostatic interactions. Under acidic conditions (pH < 6), the protonation of primary amine groups (–NH_3_^+^) facilitates strong electrostatic attraction toward anionic species like chromate (CrO_4_^2−^) and arsenate (AsO_4_^3−^) [51,61]. For cationic metals, the lone electron pairs on nitrogen and oxygen atoms coordinate with metal ions, forming stable five- or six-membered chelate rings [61,197]. Ion exchange also contributes significantly, especially when chitosan is combined with inorganic fillers such as zeolites or layered silicates, where target metal ions replace mobile counterions during the uptake process [63].

**Figure 2:**
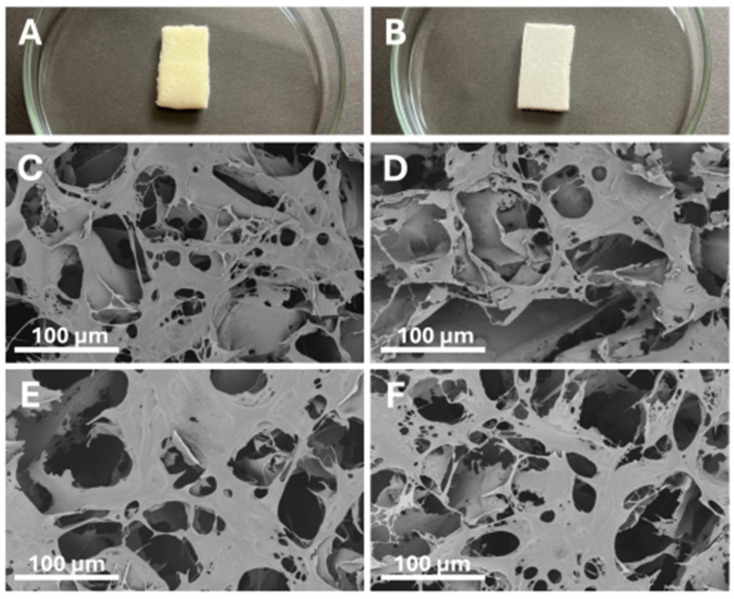
The representative photographic images of the (**A**) sponges made of microcrystalline chitosan sponge and (**B**) sponge made of microcrystalline chitosan with the addition of GO. SEM image of the outer surface of the sponge made of microcrystalline chitosan sponge with and without the addition of GO, where (**C**) microcrystalline chitosan sponge surface before sterilization, (**D**) microcrystalline chitosan sponge + GO sponge surface before sterilization, (**E**) microcrystalline chitosan sponge surface after sterilization (25 kGy), (**F**) microcrystalline chitosan sponge + GO sponge surface after sterilization (25 kGy). Magnification 1500×, presented scale bar indicates 100 µm [200].

To enhance performance, chitosan is often integrated into composite structures. Hydroxyapatite-chitosan composites leverage the high affinity of calcium phosphate for divalent cations like Pb^2+^ and Cd^2+^, while chitosan provides mechanical stability and additional binding sites [198]. Similarly, Fe_3_O_4_-chitosan magnetic composites offer an excellent adsorption capacity combined with ease of separation via external magnetic fields, enabling efficient recovery and reuse without the need for filtration [199]. Graphene oxide (GO)-chitosan composites offer ultra-high surface area and π–π stacking capabilities, improving sorption kinetics and capacity for both metallic and organic contaminants. These hybrid systems demonstrate synergistic effects, outperforming individual components in real wastewater matrices [138]. Chitosan-based biomaterials show promise for mercury removal from water. Functional amino and hydroxyl groups interact with mercury ions, allowing for effective adsorption. Raw chitosan has limits. It dissolves in acidic conditions and lacks mechanical strength. To fix this, chitosan composites with materials like graphene oxide or magnetic nanoparticles are used. For example, chitosan-graphite oxide nanocomposites remove toxic mercury ions efficiently [138]. Visualization of chitosan sponge and chitosan sponge with GO, characterized by numerous pores of various sizes, is shown in Figure 2.

Adsorption efficiency is typically evaluated using kinetic and equilibrium models. Kinetic studies commonly follow pseudo-second-order behaviour, indicating chemisorption as the rate-limiting step, while diffusion models reveal contributions from film and intraparticle transport [114,201]. Equilibrium data are best described by the Langmuir isotherm, suggesting monolayer adsorption, or the Freundlich model, which indicates heterogeneous surface binding. Maximum adsorption capacities reported in the literature vary widely, depending on the composite design, from hundreds to over 1000 mg/g, highlighting the tunability of these materials [147,202].

Regeneration and reuse are essential for economic viability. Spent chitosan composites can be eluted with dilute acids (e.g., HCl) or chelating agents (e.g., EDTA), recovering up to 80% of the initial capacity after multiple cycles. However, repeated exposure to harsh regeneration protocols may degrade the polymer matrix, necessitating robust crosslinking strategies [49,90].

A notable case study involves an EDTA-functionalized magnetic chitosan nanocomposite, designed for selective and efficient capture of Pb(II) and Cd(II) [49]. The incorporation of ethylenediaminetetraacetic acid (EDTA) moieties enhances chelation strength, while Fe_3_O_4_ nanoparticles allow magnetic retrieval. This system exhibits rapid kinetics, high selectivity even in multi-metal solutions, and effective regeneration using mild acid washes, demonstrating its potential for scalable deployment in industrial wastewater treatment [199,203].

#### 3.1.2. Dye Removal

Industrial effluents from textile, paper, and printing sectors contain persistent synthetic dyes, which are classified as anionic (e.g., Reactive Orange 16, Methyl Orange), cationic (e.g., Methylene Blue), and azo dyes (e.g., Reactive Red 120) that pose significant ecological risks due to their toxicity and resistance to degradation [49,114]. Chitosan effectively removes these dyes primarily through electrostatic adsorption, where protonated amino groups attract oppositely charged dye molecules. Anionic dyes are readily captured under acidic conditions, whereas cationic dyes require modification of chitosan’s surface charge [204].

Crosslinking agents such as glutaraldehyde, epichlorohydrin, or bisphenol A diglycidyl ether improve chemical stability and prevent dissolution in aqueous media [51]. Functionalization strategies, including quaternization, introduce permanent positive charges, thereby extending the applicability of chitosan to neutral and alkaline environments. Quaternized chitosan derivatives exhibit enhanced affinity for anionic dyes regardless of pH, offering operational flexibility [205].

Chitosan is processed into various forms, including composite films, beads, and hydrogels, to optimize contact area and mass transfer. Beads prepared via dropwise gelation exhibit high reusability in fixed-bed columns, while hydrogels offer swelling-controlled release and enhanced accessibility of functional groups [206]. Films incorporating graphene oxide or clay minerals exhibit superior mechanical strength and barrier properties, making them suitable for membrane-based filtration [128].

Despite their efficacy, challenges remain. High ionic strength and competing anions (e.g., SO_4_^2−^, Cl^−^) can interfere with dye adsorption, reducing efficiency [207]. Additionally, some systems suffer from slow kinetics due to limited pore diffusion, particularly in dense matrices. Addressing these limitations requires tailored porosity engineering and the use of stimuli-responsive designs [208].

#### 3.1.3. Organic Pollutant Adsorption

Beyond metals and dyes, chitosan composites are increasingly used to remove emerging organic pollutants, including pharmaceuticals, pesticides, and endocrine disruptors (e.g., bisphenol A, ibuprofen, carbamazepine). The dominant mechanisms include π-π stacking between aromatic rings in contaminants and graphitic domains in carbonaceous supports, as well as hydrogen bonding involving –OH and –NH_2_ groups [209].

Graphene oxide-chitosan composites are particularly effective due to GO’s large surface area and oxygen-containing functional groups, which enhance dispersion and interaction with polar organics [210]. In more advanced systems, molecularly imprinted polymers (MIPs) based on chitosan templates create selective recognition sites for specific analytes, mimicking the specificity of antibodies [211]. However, challenges persist in achieving high selectivity in complex mixtures and ensuring complete regeneration without template leaching.

### 3.2. Waste Management

#### 3.2.1. Biodegradable Packaging

**Figure 3:**
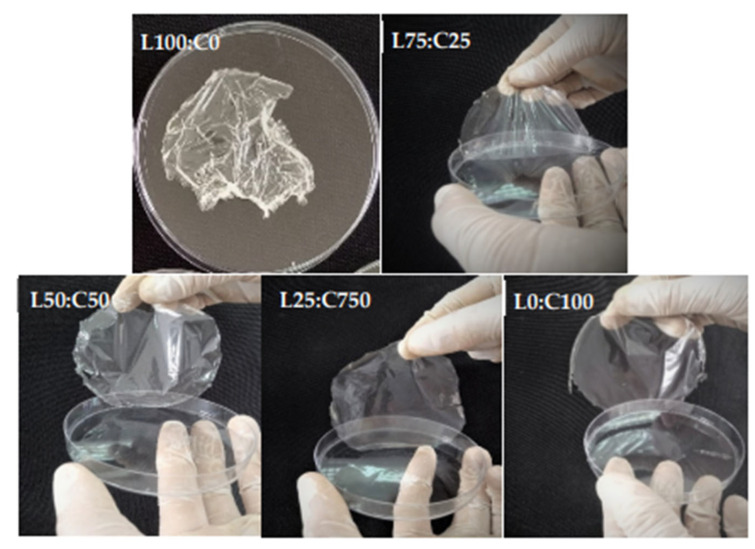
Visual appearance of chitosan-levan films [215].

Chitosan-based films and coatings represent a sustainable alternative to petroleum-derived plastics in food packaging. Their inherent antimicrobial activity, film-forming ability, and biodegradability make them ideal for preserving perishable goods [212]. These films act as barriers against moisture, oxygen, and microbial contamination, thereby extending shelf life. However, pure chitosan films exhibit high hydrophilicity, limiting their moisture resistance [213,214]. This drawback is mitigated by blending with hydrophobic polymers or incorporating nanofillers such as silver nanoparticles, clay minerals, and lignin, which improve tensile strength, reduce water vapor permeability, and impart additional bioactivity [29]. As a progressive composite for these applications, chitosan-levan films (Figure 3) seem to be the most suitable choice, offering favourable mechanical performance, as well as antioxidant and antimicrobial properties.

Silver nanoparticles exhibit potent antibacterial effects, while layered silicates, such as montmorillonite, enhance thermal stability and gas barrier properties. Lignin, a natural polyphenolic compound, provides antioxidant functionality and UV protection [216]. Despite promising lab-scale results, commercial adoption faces hurdles related to cost, scalability, and regulatory approval. Large-scale production remains constrained by inconsistent raw material quality and energy-intensive processing methods. Nevertheless, pilot-scale implementations in fruit and seafood packaging show tangible benefits, supporting gradual market integration [217].

#### 3.2.2. Composting Additives

In solid waste valorization, chitosan serves as a composting additive that accelerates the decomposition of organic matter and reduces environmental emissions [11]. It stimulates microbial activity by acting as a nitrogen-rich substrate for beneficial bacteria and fungi [218]. Moreover, chitosan helps retain nitrogen in compost by forming complexes with ammonium ions, minimizing volatilization losses. Field trials indicate that chitosan-coated compost amendments reduce greenhouse gas emissions, particularly methane and nitrous oxide, by modulating microbial pathways [219].

Optimal dosages range between 0.5% and 2% *w*/*w*, beyond which inhibitory effects may occur. Long-term soil applications demonstrate improved aggregate stability and nutrient retention, although excessive use may alter the soil microbiota [95,220,221]. Sustainable sourcing from seafood waste further enhances the circular economy appeal of chitosan in waste-to-resource strategies.

### 3.3. Soil Remediation

#### 3.3.1. Heavy Metal Immobilization

In contaminated soils, chitosan facilitates the immobilization of heavy metals through complexation, precipitation, and ion exchange. Reducing metal bioavailability prevents plant uptake and groundwater leaching. Biochar/chitosan hybrids are particularly effective, as they combine the high surface area and cation exchange capacity of biochar with the chelating functionality of chitosan [158]. These composites stabilize metals like Pb, Cd, and Cu in rhizosphere zones, promoting safer agricultural practices [95].

Field-scale applications demonstrate reduced metal mobility over extended periods, though long-term stability under varying pH and redox conditions requires further study. Integration into soil amendments offers a low-cost, eco-friendly strategy for rehabilitating mining-affected lands [222].

#### 3.3.2. Pesticide Remediation

For pesticide-contaminated soils, chitosan functions via sorption mechanisms, trapping organophosphates and chlorinated compounds through hydrogen bonding and hydrophobic interactions [223]. Chitosan-clay hybrids increase sorption capacity by expanding interlayer spacing and providing additional binding sites. Some formulations incorporate enzymes, such as laccase or peroxidase, which enable the enzymatic degradation of pesticides into less toxic metabolites [224]. Despite these advantages, real-world implementation is hindered by variability in soil composition, moisture content, and microbial interference, necessitating site-specific optimization.

### 3.4. Air Purification

**Figure 4:**
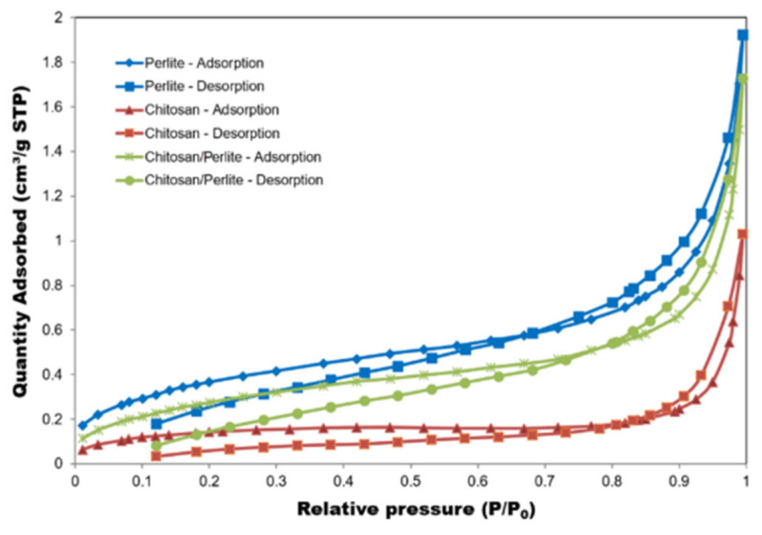
Nitrogen adsorption/desorption isotherms for perlite, chitosan, and chitosan/perlite composite [225].

Chitosan is playing a growing role in capturing pollutants from the air, targeting volatile organic compounds (VOCs), NO_x_, and SO_2_. Chitosan-MOF composites utilize the high porosity and tunable pores of metal–organic frameworks to adsorb gaseous pollutants selectively [40,138]. Additionally, activated carbon derived from chitosan pyrolysis offers a green route to porous adsorbents with high surface areas. These materials can be integrated into HVAC filters or wearable masks for indoor and personal air purification [59]. While promising, scalability and cost-effectiveness remain key considerations for widespread deployment. Another example is chitosan-perlite composite, which was found to be much more effective for nitrogen adsorption over pure chitosan [225], as demonstrated in Figure 4.

### 3.5. Air Filtration

Chitosan is naturally positively charged, giving it strong antimicrobial properties. It can attach to and disrupt the membranes of airborne microorganisms. This trait has been used in food packaging, where chitosan-polymer composites help control microbial growth in the surrounding air [226,227]. Electrospun chitosan nanofibers, often blended with cellulose or enhanced with zinc oxide nanoparticles, form porous, high-surface-area mats that capture and inactivate bioaerosols. Studies show chitosan-ZnO-nisin coatings can suppress spoilage organisms in cheese packaging [228,229]. The amine and hydroxyl groups on chitosan also allow it to interact with polar volatile organic compounds, suggesting it could help adsorb volatile organic compounds. Its nanofibrous structure offers low air resistance and efficient particle capture, making it well-suited for air filtration [230]. While direct use in heating, ventilation, air conditioning, or industrial systems has not been widely studied, research on smart chitosan composites that release antimicrobials like thymol or liquid smoke shows potential for self-sanitizing filters. Being biodegradable, easy to modify, and compatible with green techniques like electrospinning, chitosan nanofibers offer a sustainable solution for improving air quality in healthcare, food storage, and indoor environments [231].

### 3.6. Antimicrobial Activity

**Figure 5:**
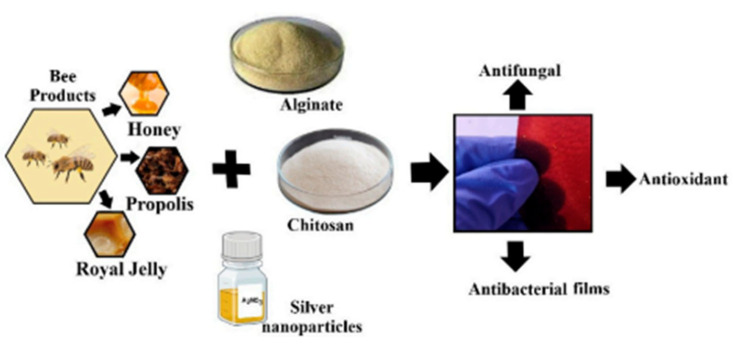
Schematic interpretation of film preparation based on chitosan, alginate, silver nanoparticles, and natural bee products [231].

Chitosan is widely used in surface coatings for textiles, medical devices, and food packaging due to its intrinsic antimicrobial properties [232]. Mechanisms include the disruption of microbial cell membranes through electrostatic interactions with negatively charged phospholipids and the inhibition of DNA replication after cellular internalization [233,234]. Chemical modifications such as grafting N-halamines or quaternary ammonium salts enhance biocidal activity. However, concerns about nanoparticle leaching and the development of microbial resistance warrant careful formulation and lifecycle assessment [235,236]. A particularly interesting substrate is the composite based on chitosan, alginate, and silver nanoparticles, which is enriched with natural bee products (Figure 5). Such materials have interesting antioxidant, antifungal, and antibacterial properties [237].

### 3.7. Oil Spill Cleanup

For marine oil spills, modified chitosan aerogels and foams serve as highly absorbent materials. Hydrophobically modified chitosan exhibits selective uptake of non-polar hydrocarbons while repelling water [162,238]. These porous structures offer high absorption capacities (up to 30 times their weight), reusability through squeezing or solvent extraction, and good mechanical durability [146]. Challenges include maintaining performance under turbulent sea conditions and preventing secondary pollution upon degradation [239,240].

### 3.8. Other Environmental Applications

Chitosan finds diverse uses in agricultural films, erosion control mats, biosensors, controlled-release fertilizers, desalination membranes, and gas separation membranes [20]. Its biodegradability supports temporary ground cover solutions, while its responsiveness enables innovative delivery systems. In membrane technology, chitosan-based thin-film composites show promise in forward osmosis and CO_2_/N_2_ separation, contributing to clean energy and water security [3].

## 4. Discussion

**Figure 6:**
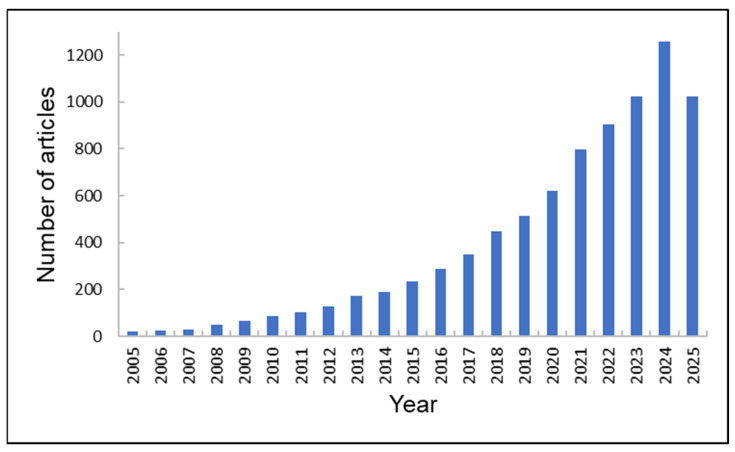
The number of scientific articles on environmental applications of chitosan retrieved from ISI WoS. The value for 2025 is by the end of August.

The environmental applications of chitosan and its derivatives have emerged as a cornerstone in the development of green, biodegradable, and cost-effective solutions for pollution mitigation [160]. Figure 6 represents the number of scientific papers published annually in the last 20 years, where evident increase can be observed.

As evidenced by the body of research analyzed in this review, chitosan-based materials, particularly composites and chemically modified forms, demonstrate exceptional performance in the removal of heavy metals, organic dyes, pharmaceutical residues, and other contaminants from aqueous environments [9]. However, while laboratory-scale studies consistently report high adsorption capacities (often exceeding 100–200 mg/g for metals like Pb(II), Cd(II), and Cr(VI)), the transition from benchtop innovation to real-world implementation remains hindered by several interrelated scientific, engineering, and economic barriers. A central theme across the reviewed literature is the strategic modification of native chitosan to overcome its inherent limitations: solubility at low pH, poor mechanical strength, and limited selectivity under competitive ion conditions [69]. The integration of chitosan with inorganic nanoparticles (e.g., TiO_2_, ZnO, Fe_3_O_4_), clays (e.g., montmorillonite), carbon nanomaterials (graphene oxide, CNTs), or biopolymers (cellulose, alginate) significantly enhances stability, surface area, and functional group density [110]. For instance, magnetic chitosan composites (e.g., Fe_3_O_4_/chitosan) not only improve separation efficiency but also facilitate regeneration and reuse, which is critical for circular economy models [158]. Previous studies highlight that hybridization with metal oxides introduces additional adsorption mechanisms, including electrostatic attraction, ion exchange, and surface complexation, thereby broadening the range of treatable pollutants. Moreover, chemical derivatization such as quaternization, crosslinking with glutaraldehyde, or grafting with functional groups (–NH_2_, –COOH, –SH) alters the protonation behavior and binding affinity of chitosan [73,77]. Quaternized chitosan derivatives, for example, remain positively charged across a wide pH range, enabling effective anion removal (e.g., fluoride, arsenate) even in acidic conditions where unmodified chitosan dissolves [46,73]. This is particularly relevant for arsenic remediation, where recent studies report enhanced As(V) uptake via ligand exchange with surface hydroxyl groups on chitosan-TiO_2_ composites [51,155]. Kinetic and isotherm modeling dominate the analytical framework in most studies, with pseudo-second-order kinetics and Langmuir isotherms frequently cited as best fits. While these models suggest chemisorption and monolayer adsorption, their overuse without complementary spectroscopic validation (e.g., XPS, FTIR, EXAFS) risks oversimplification [49]. Several papers rely solely on model fitting without probing actual binding mechanisms, leading to potentially misleading conclusions about adsorption pathways. For example, attributing Cr(VI) removal entirely to electrostatic attraction overlooks the well-documented reduction of Cr(VI) to Cr(III) by amine groups, followed by coordination of the reduced species, a dual mechanism confirmed in multiple spectroscopic studies [51,203]. The removal of Cr(VI) by chitosan-based materials proceeds through a well-documented three-step mechanism: adsorption, reduction, and complexation. In acidic conditions (pH 2–4), the amino groups (–NH_2_) of chitosan become protonated to –NH_3_^+^, enabling strong electrostatic adsorption of anionic Cr(VI) species (e.g., HCrO_4_^−^ or CrO_4_^2−^) onto the polymer surface. This initial adsorption concentrates Cr(VI) at the active sites. Subsequently, the amino groups act as electron donors, reducing highly toxic and mobile Cr(VI) to less toxic and less soluble Cr(III). This redox reaction is supported by XPS analyses showing the presence of both Cr(VI) and Cr(III) on spent adsorbents, confirming the reduction step. Finally, the generated Cr(III) ions form stable coordination complexes with the remaining unprotonated amino and hydroxyl groups of chitosan, effectively immobilizing the metal. This adsorption–reduction–complexation sequence not only removes Cr(VI) from solution but also detoxifies it, making chitosan a dual-functional material for chromium remediation. The process is enhanced in composites (e.g., with graphene oxide, Fe_3_O_4_, or ZnO), where additional functional groups or metal centers can facilitate electron transfer and improve stability [51,203]. Furthermore, the majority of isotherm experiments are conducted using single-solute systems, which poorly represent real wastewater matrices containing multiple competing ions, natural organic matter, and suspended solids. Only a minority of the reviewed studies evaluate performance in multi-pollutant or real industrial effluents, revealing significant capacity drops due to fouling or ion competition. This discrepancy between idealized and realistic conditions underscores the need for more environmentally relevant testing protocols. Despite claims of “reusability,” many studies report only 3–5 regeneration cycles, often with >20% capacity loss by the final cycle [90,118,195]. The use of strong acids (HCl) or bases (NaOH) for desorption raises concerns about long-term structural integrity and secondary pollution. In some cases, the regeneration process itself generates hazardous waste streams, undermining the environmental benefits of the original adsorbent [110]. Additionally, few studies address the end-of-life fate of spent chitosan composites. While chitosan is inherently biodegradable, the presence of non-biodegradable components (e.g., synthetic polymers, metal nanoparticles) may inhibit decomposition or lead to nanoparticle leaching during disposal. A truly sustainable chitosan composite must be evaluated not only on adsorption efficiency but also on lifecycle impacts, including raw material sourcing (e.g., shrimp vs. fungal chitosan), energy consumption during synthesis, reusability, and final disposal. Such parameters are compared for various systems in following Table 2.

The shift toward waste-derived chitosan (e.g., from crustacean shells, squid pens, or fungal biomass) aligns with circular economy principles and reduces reliance on finite resources. However, the scalability of such green synthesis routes remains a challenge, particularly in regions lacking seafood processing infrastructure. The overwhelming majority of research remains confined to batch adsorption experiments at a small scale (<100 mL) [145]. Continuous flow systems, column studies, and pilot-scale trials are underrepresented, despite their relevance to industrial water treatment. Only a few studies explore chitosan-based membranes or fixed-bed reactors, which are more suitable for large-scale deployment [160]. Even then, issues such as membrane fouling, pressure drop, and mechanical degradation under prolonged operation are rarely addressed in depth. Moreover, the physical form of the material, bead, film, membrane, or powder, affects handling, flow dynamics, and applicability. For instance, chitosan films show promise in air filtration and volatile organic compound (VOC) capture, while magnetic nanoparticles enable rapid separation in slurry systems [135]. Yet, the lack of standardized performance metrics across different formats makes comparative evaluation difficult. An analysis of funding sources reveals a strong concentration of research in Asia (China, India, Malaysia, Iran) and Europe, with significant support from national science foundations and regional development programs (e.g., NSFC, YUTP, EU-NextGenerationEU). This reflects strategic investment in green materials science but also highlights disparities in global research capacity. Nearly 10% of the reviewed studies report no external funding, suggesting that some innovations arise from under-resourced institutions, potentially limiting reproducibility or scale-up potential. Another critical gap is the inconsistent reporting of data availability and experimental details. While open-access publishing has increased transparency, many authors restrict raw data access to “upon request,” hindering meta-analyses and independent validation. Similarly, detailed synthesis protocols, especially for nanocomposites, are often omitted, making replication challenging. The future of chitosan-based environmental materials lies not merely in incremental performance improvements but in the development of intelligent, multifunctional systems. Emerging trends include stimuli-responsive composites (pH-, temperature-, or light-sensitive), self-cleaning photocatalytic hybrids (e.g., chitosan-TiO_2_), and integration with digital monitoring (IoT-enabled sensors). Additionally, combining adsorption with catalytic degradation (e.g., Fenton-like systems using Fe-chitosan composites) offers a pathway beyond mere pollutant sequestration toward complete mineralization. Finally, policy alignment and regulatory acceptance are essential for widespread adoption. Standardization of testing protocols, certification of biodegradability, and lifecycle assessment frameworks will be crucial in transitioning chitosan composites from academic curiosities to commercially viable environmental technologies.

## 5. Limitations and Future Perspectives

Despite the significant advancements in the development and application of chitosan-based materials for environmental remediation, several critical limitations hinder their widespread industrial adoption and long-term sustainability. These challenges span the entire lifecycle of chitosan products from raw material sourcing and synthesis to performance under real-world conditions, regeneration, and end-of-life management. Addressing these constraints is essential for transitioning chitosan technology from laboratory-scale innovation to large-scale, practical implementation.

One of the primary limitations lies in the scalability of synthesis methods. Many high-performance chitosan composites are fabricated using energy-intensive, batch-dependent techniques such as freeze-drying, cryostructuring, or layer-by-layer assembly [22]. These processes are challenging to scale up due to high operational costs, low throughput, and the requirement for specialized equipment such as lyophilizers or supercritical CO_2_ dryers. Furthermore, the use of toxic crosslinking agents, such as glutaraldehyde, enhances mechanical stability but raises environmental and health concerns, particularly regarding the disposal of residual chemicals and spent adsorbents [52]. Although greener alternatives, such as genipin or enzymatic crosslinking, are being explored, their high costs and limited availability restrict their broad application.

Another persistent challenge is the instability of native chitosan under neutral and alkaline pH conditions, where it loses its solubility and cationic character due to the deprotonation of amino groups [69]. This significantly limits its effectiveness in natural water systems and industrial effluents that operate outside the acidic range. While chemical modifications, such as quaternization or carboxymethylation, can extend the functional pH range, these derivatization processes often require multiple reaction steps, thereby increasing complexity and production costs. Moreover, excessive crosslinking or functionalization may reduce the accessibility of active binding sites, thus compromising adsorption capacity and kinetics.

The effectiveness of chitosan modification strategies depends on the specific application and environmental conditions. Crosslinking improves stability in acidic environments, making it ideal for wastewater treatment at low pH, though it may reduce adsorption capacity. Grafting introduces selective binding sites, enhancing metal uptake in complex matrices. Composite formation with nanoparticles like Fe_3_O_4_ or TiO_2_ enables magnetic separation and photocatalytic degradation, suitable for multifunctional systems under light exposure. For anionic pollutants, quaternization provides permanent positive charges, ensuring high adsorption across a broad pH range. Green modifications using citric acid or genipin offer improved biocompatibility and reusability, favoring sustainable applications. Thus, the optimal strategy should align with operational needs: crosslinking for harsh conditions, grafting for selectivity, nanocomposites for combined removal-degradation, and ionic modification for anion capture.

The mechanical durability of chitosan-based materials under dynamic environmental conditions is also a concern. In continuous flow systems, such as fixed-bed columns or membrane filtration units, repeated swelling and deswelling cycles can lead to structural fatigue, particle disintegration, and loss of porosity [22]. This issue is particularly evident in hydrogels and aerogels, which demonstrate excellent adsorption performance in batch experiments but struggle with mechanical resilience in real-world applications. Additionally, fouling caused by organic matter, colloidal particles, or microbial biofilms can block active sites and reduce efficiency over time, necessitating frequent cleaning or replacement. However, some promising chitosan composite systems are already recognized. For example, chitosan/TiO_2_ membrane exhibits a much lower degree of swelling than pure chitosan in water and buffers [249], as seen in Table 3.

Regeneration and reusability, while theoretically feasible, present practical and ecological trade-offs. Although many chitosan composites can be regenerated using acidic or basic eluents, the repeated use of strong chemicals may degrade the polymer matrix, leach functional components, or generate secondary waste streams that require treatment. For instance, regenerating metal-loaded composites with acids produces concentrated metal-laden effluents that must be managed responsibly. Furthermore, the economic feasibility of regeneration depends on balancing recovery efficiency with operational costs, including water and energy consumption during washing and drying cycles [90].

From a lifecycle sustainability perspective, conventional chitosan production via chemical demineralization and deacetylation generates substantial amounts of acidic and alkaline wastewater, contributing to environmental pollution [160]. Although enzymatic and microbial extraction methods offer a more sustainable alternative by reducing chemical usage and energy input, they are currently less efficient and more time-consuming than traditional processes. Scaling up these green synthesis routes requires further optimization of microbial strains, enzyme immobilization techniques, and bioreactor designs to achieve industrial throughput and cost-effectiveness.

Looking ahead, the future of chitosan-based environmental technologies lies in integrating advanced material design, digital innovation, and principles of the circular economy. The development of multifunctional composites such as magnetic, photocatalytic, or stimuli-responsive systems will enable not only pollutant capture but also degradation, sensing, and controlled release. For example, integrating chitosan with metal–organic frameworks (MOFs) or graphene oxide can enhance selectivity and catalytic activity, while the use of magnetic nanoparticles facilitates easy separation and reuse [9].

Artificial intelligence (AI) and machine learning (ML) are poised to revolutionize the field by enabling predictive modeling of adsorption behavior, optimizing composite formulations, and supporting real-time monitoring in treatment plants [186]. Digital twins and blockchain-based traceability systems could ensure transparency across the supply chain, further driving the sustainable adoption of chitosan-based materials for environmental remediation.

## 6. Conclusions

The escalating environmental challenges of the 21st century demand innovative, sustainable, and practical solutions. This review comprehensively examines the potential of chitosan, a naturally abundant and biodegradable biopolymer, along with its numerous derivatives and composites, as a cornerstone material for addressing these pressing issues. Chitosan’s unique chemical structure, featuring reactive amino and hydroxyl groups, provides the foundation for its remarkable versatility in interacting with a broad spectrum of pollutants, including heavy metals, dyes, organic contaminants, and microorganisms. However, the inherent limitations of native chitosan, such as poor solubility outside acidic conditions, limited mechanical strength, and susceptibility to swelling, necessitate strategic modifications to realize its full potential in practical applications.

Modern modification strategies, encompassing chemical derivatization (crosslinking, grafting, quaternization, functionalization), physical structuring (forming beads, films, aerogels, membranes), and, most significantly, composite formation, have proven highly effective. These approaches allow for the precise tailoring of chitosan-based materials to enhance stability, selectivity, and efficiency. The creation of composites by integrating chitosan with inorganic fillers (silica, clays, metal oxides), carbonaceous materials (graphene oxide, biochar, CNTs), magnetic nanoparticles (Fe_3_O_4_), other biopolymers (alginate, lignin), and even metal–organic frameworks (MOFs) has led to the development of hybrid materials with synergistic properties that often surpass the sum of their components. These advanced materials can be engineered for specific functions, such as targeted adsorption, catalytic degradation, efficient membrane filtration, or effective flocculation.

The mechanisms underlying pollutant removal by chitosan-based systems are multifaceted and often work in concert. Adsorption, driven by electrostatic interactions, complexation, ion exchange, and hydrogen bonding, remains the dominant mechanism. However, the integration of catalytic functionalities enables the active degradation of pollutants, while the material’s film-forming ability and surface charge properties make it suitable for antifouling membrane applications. Furthermore, its cationic nature allows it to function effectively as a green flocculant. This mechanistic diversity highlights the adaptability of chitosan platforms in addressing complex environmental matrices.

Despite the overwhelming success demonstrated in laboratory and pilot-scale studies, the transition from bench-scale innovation to widespread real-world implementation faces several practical challenges. Issues related to the scalability of often energy-intensive fabrication methods (like lyophilization), ensuring long-term stability and performance under variable and harsh environmental conditions, developing efficient and environmentally benign regeneration protocols, and conducting thorough lifecycle sustainability assessments must be addressed. Economic viability is closely linked to the cost-effective sourcing of raw materials, favouring the use of unrefined seafood waste over purified chitosan. Embracing green synthesis routes, such as enzymatic processing and microbial fermentation, is crucial for aligning production with sustainability goals.

Looking ahead, the field is being shaped by exciting emerging trends. The integration of chitosan technology within circular economy models is paramount, transforming seafood processing waste into high-value materials and designing end-of-life strategies for spent composites that involve metal recovery or composting. The valorisation of seafood waste using green extraction techniques further enhances the sustainability profile. Perhaps most significantly, the infusion of digital innovation, particularly artificial intelligence (AI) and machine learning (ML), is revolutionizing the design, optimization, and operational monitoring of chitosan-based systems. These tools enable predictive modelling, accelerate material discovery, optimize process parameters, and facilitate real-time control, paving the way for intelligent and efficient environmental solutions.

Thus, chitosan and its derivatives/composites represent an up-and-coming class of materials for sustainable environmental protection. The extensive research and development efforts highlighted in this review demonstrate the significant progress made in overcoming the limitations of native chitosan and engineering high-performance materials tailored for specific environmental applications. From water treatment and soil remediation to air purification and waste management, the versatility of chitosan-based systems is evident. While challenges remain in scaling up production, ensuring robustness, and achieving full economic competitiveness, the convergence of advanced material science, green chemistry, circular economy principles, and digital optimization presents a clear pathway forward. Continued research focusing on practical implementation, lifecycle assessment, and the integration of innovative technologies will be essential. By addressing these aspects, chitosan-based technologies can move beyond laboratory curiosities to become integral components of scalable, intelligent, and ecologically responsible solutions, playing a vital role in safeguarding the environment for future generations.

## Figures and Tables

**Table 1:** Comparative life cycle impact of chitosan-based materials based on source and modification method.

Impact Category	Crustacean Chitosan + Chemical Modification	Fungal Chitosan + Enzymatic/Green Modification
Raw Material Source	Shrimp/crab shells (waste stream, seasonal, geographically limited)	*Aspergillus niger*, *Mucor rouxii* (cultivated, consistent supply)
Deacetylation Process	Concentrated NaOH, 90–120 °C, 3–6 h	Enzymatic (chitin deacetylase), 40–60 °C, mild pH
Energy Consumption	High (8–12 kWh/kg)—due to high-temperature alkaline treatment	Medium (5–7 kWh/kg)—lower thermal demand and reduced chemical load
Carbon Footprint (CO_2_-eq)	~5–7 kg CO_2_/kg—high emissions from NaOH production and waste treatment	~2–3 kg CO_2_/kg (~30–40% lower)—lower chemical use, biodegradable reagents
Chemical Waste	High—alkaline effluent, high chemical oxygen demand, acid washes for regeneration	Low—biodegradable enzymes, citric acid, or genipin; less hazardous byproducts
Water Use	High—multiple washing steps to remove proteins, minerals, and residual alkali	Moderate to low—closed-loop fermentation possible, less washing required
Modification Method	Glutaraldehyde, epichlorohydrin—toxic, non-biodegradable	Moderate to low—closed-loop fermentation possible, less washing required
Reusability	Moderate (3–5 cycles, >20% capacity loss)—structural degradation from acid/base regeneration	Moderate to high—improved stability with green crosslinking, less leaching
End-of-Life Biodegradability	High (pure chitosan); reduced in composites with synthetic polymers or nanoparticles	High; potentially enhanced by cleaner synthesis and absence of persistent toxicants
Nanoparticle Leaching Risk	Moderate to high—Fe_3_O_4_, TiO_2_, ZnO in composites may leach under acidic conditions or after reuse	Similar risk if nanomaterials are used, but lower if green composites are designed

**Table 2:** Comparative performance of chitosan-based composites in terms of adsorption capacity, reusability, and stability.

Adsorbent	Target Pollutant	Max. Adsorption Capacity (mg/g)	Regeneration Cycles	Remaining Efficiency After Last Cycle (%)	Eluent Used	Key Stability Features	Ref.
MIL-53(Fe)/Chitosan hydrogel spheres	Congo Red (CR)	590.8	3	~85%	ethanol	High porosity, good structural integrity	[241]
MWSBC-0.5 (magnetic straw-based composite)	Cr(VI)	80.79	7	78.6%	HCl	Magnetic separation; stable at pH 5	[242]
PEI-functionalized chitosan hydrogel	Pb(II)	~100	4	~81%	1 M HCl	Swelling-resistant; reusable	[243]
Chitosan-MOF composite	Pb(II)	98	5	>80%	0.5 M Na_2_SO_4_	Good cyclic performance	[90]
CTS-STPP-MS	Orange II	948	5	97.85	NaOH	Easy separation, regenerable	[49]
PVA-CS aerogel	Cu(II)	111.85	3	N/A	0.1 M Na_2_EDTA	Robust film structure	[244]
Ch-Fe composite	As(V)	16.1	2	N/A	N/A	Enhanced Fe-NP immobilization	[245]
Coffee-chitosan (50:50)	Methylene Blue (MB)	75.76	Not tested	N/A	N/A	Natural, low-cost composite	[246]
HAp/CTS composite	Cd(II)	126.58	10	56.5	DI water	Thermally stable; good ion exchange	[247]
CS/n-HAp composite	Cd(II)	122	Not tested	N/A	N/A	Biocompatible; stable in aqueous media	[248]

**Table 3:** Degree of swelling of chitosan membrane and chitosan/TiO_2_ composite membrane in water and different aqueous buffer solutions at pH 6, 7, and 8 [249]. The same letters denote statistical similarity of samples with variation in the solution. Data expressed as mean and standard deviation, with 95% confidence according to Tukey’s test.

Sample	Degree of Swelling (%)			
	H_2_O	pH 6	pH 7	pH 8
Chitosan	481.59 ± 74.26 ^a^	138.20 ± 6.51 ^b^	171.17 ± 40.92 ^b^	180.50 ± 38.53 ^b^
Composite	376.25 ± 8.31 ^a^	115.42 ± 10.11 ^c^	158.81 ± 7.77 ^b^	131.20 ± 5.95 ^b^

## Data Availability

No new data were created.

## Abbreviations

The following abbreviations are used in this manuscript:

DD	Degree of deacetylation
MW	Molecular weight
CMCS	Carboxymethyl chitosan
ECH	Epichlorhydrin
BADGE	Bisphenol A diglycidyl ether
PEI	Polyethyleneimide
ChNS	Chitosan-nanosilica composites
ChSG	Chitosan-silica gel composites
GO	Graphene oxide
CNTs	Carbon nanotubes
NS	Nanosilica
SG	Silica gel
GA	Glutaraldehyde
CBZ	Carbamazepine
CIP	Ciprofloxacin
SEM	Scanning electron microscopy
PDA	Polydopamine
TPP	Tripolyphosphate
Car/CSAs	Chitosan/carbon ratio in aerogel composites
MB	Methylene blue
CSGO	Chitosan and graphene oxide membrane
MMMs	Mixed matrix membranes
MOFs	Metal–organic frameworks
LCA	Lifecycle analysis
AI	Artificial intelligence
ML	Machine learning
ANFIS	Adaptive neuro-fuzzy inference systems
ANNs	Artificial neural networks
RSM	Response surface methodology
EDTA	Ethylenediaminetetraacetic acid
MIPs	Molecularly imprinted polymers
VOCs	Volatile organic compounds
